# The pressure gradient for venous return and its derivatives are ambiguous measures

**DOI:** 10.1186/s40635-024-00692-x

**Published:** 2024-11-11

**Authors:** Jon-Emile S. Kenny, Per Werner Moller

**Affiliations:** 1 Health Sciences North Research Institute, Sudbury, ON Canada; 2 Flosonics Medical, Toronto, ON Canada; 3 Department of Anesthesia, SV Hospital Group, Institute of Clinical Sciences at the Sahlgrenska Academy, University of Gothenburg, Gothenburg, Sweden

Editor:

Aneman and colleagues have published an exploratory, post-hoc analysis of the NEUROPROTECT study [1]—a randomized clinical trial comparing goal-directed hemodynamic therapy to achieve higher mean arterial pressure (MAP) in comatose survivors of cardiac arrest. The authors used hemodynamic data to calculate the mean systemic pressure analogue (*P*_MSA_) [2]. Along with the *P*_MSA_, right atrial pressure (*P*_RA_), cardiac output (CO) and MAP were used to quantify variables relevant to venous return physiology [3] which were compared between those patients randomized to goal-directed hemodynamic therapy (i.e., higher MAP) and usual care.

The use of a cardiovascular model incorporating venous return physiology allowed the authors to describe several interesting differences between the two groups. Nevertheless, we highlight for the casual reader that two of the measures—the cardiac efficiency, *E*_*h*_ and volume efficiency *E*_vol_—have an important caveat:

$$E_{h} = \frac{{P_{{{\text{MSA}}}} - P_{{{\text{RA}}}} }}{{P_{{{\text{MSA}}}} }}\;{\text{and}}\;E_{{{\text{vol}}}} = \frac{{\left( {P_{{{\text{MSA}}}} - P_{{{\text{RA}}}} } \right)_{\Delta } }}{{P_{{{\text{MSA}}\Delta }} }}$$

Both of these derived measures primarily relate to changes occurring along the *x*-axis of the Guyton diagram. Interpretation of *E*_*h*_ and *E*_vol_ in isolation, ignoring simultaneous changes in flow and/or resistances, comes with the risk of missing important physiological behavior. Accordingly, without reference to flow (on the *y*-axis), both *E*_*h*_ and *E*_vol_ are grounded on the critical assumption that the resistance to venous return (*R*_VR_) is constant (see Fig. 1A) [3]. Given that *E*_*h*_ and *E*_vol_ are confounded by *R*_VR_, these measures are, by themselves, ambiguous.

**Fig. 1:**
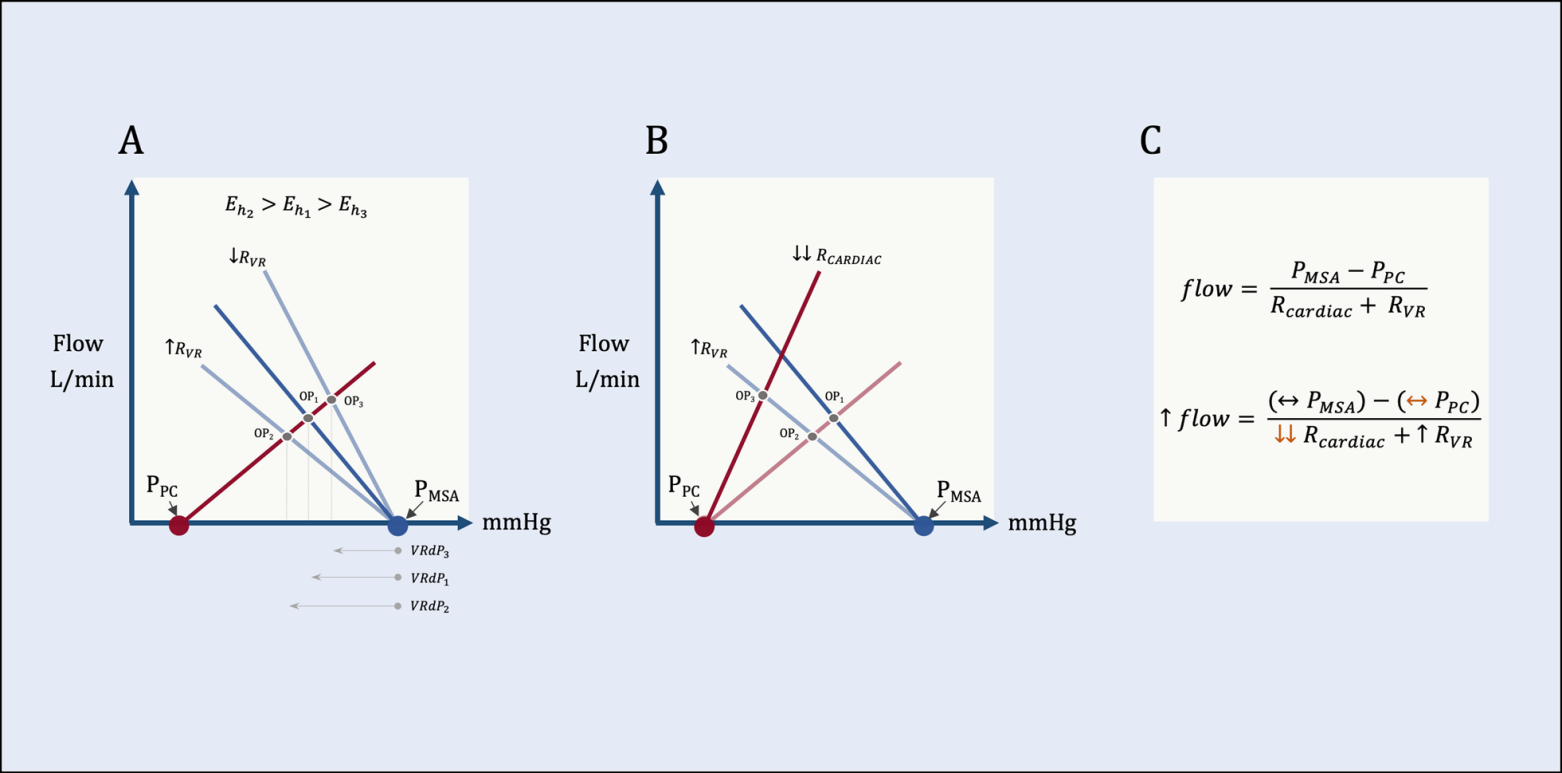
Pressure gradient for venous return (VRdP) in the context of the geometrical model. **A** Operating point 1 (OP_1_) represents baseline at the intersection of cardiac function (red line) and venous return (dark blue line). Decreasing the resistance to venous return (*R*_VR_) leads to operating point 3 (OP_3_) which shrinks the VRdP and, therefore, *E*_*h*_ and *E*_vol_ despite stable cardiac function. Increasing the R_VR_ increases VRdP (OP_2_) and, therefore, *E*_*h*_ and *E*_vol_. **B** Graphical mapping of the 10–15-h mark of the NEUROPROTECT results. Increased VRdP with increased R_VR_ with increased CO. **C** Geometrical model for the 10–15-h mark of NEUROPROTECT. There was little difference in *P*_MSA_ (black, horizontal arrow) and no reason to believe that there would be a difference in pericardial/intrathoracic pressure (*P*_PC_) between the groups (orange, horizontal arrow). There was a measured increase in blood flow and *R*_VR_ (black upwards arrows). From this, we can deduce improved cardiac function (i.e., decreased *R*_cardiac_). Black arrows represent measured variables, and orange arrows are inferred

With regard to E_h_, this is illustrated in the data presented by Aneman and colleagues at the 10–15-h mark. At this point, the pressure gradient for venous return (i.e., *P*_MSA_ − *P*_RA_ = VRdP) shows the largest difference between the usual care and intervention (i.e., higher MAP) arms with the latter having the greater VRdP. With only these data, we cannot know if the increased *E*_*h*_ during this time is truly due to improved cardiac function, especially because *R*_VR_ rose in the intervention arm during this time. Could these data represent ‘*E*_*h*2_’ in Fig. 1A? It is only when we see greater absolute CO in the intervention arm during this time that we definitively conclude improved cardiac function, or a decrease in ‘cardiac resistance’ as recently described (Fig. 1B, C) [4].

Importantly, the same assumptions apply to *E*_vol_ which is calculated as the change in the VRdP relative to change in *P*_MSA_. This is especially important when *E*_vol_ is measured in response to a fluid bolus. Guyton noted decreased *R*_VR_ following IV fluid [3]; similarly, Monge-Garcia and colleagues [5] and Aneman et al. in the NEUROPROTECT study observed decreased arterial load with fluids (i.e., decreased arterial elastance, *E*_*a*_). As arterial load/resistance is a part of *R*_VR_, IV fluids might, therefore, decrease the VRdP and *E*_*h*_ and *E*_vol_ (e.g., moving from OP_1_ to OP_3_ in Fig. 1A). However, *E*_vol_ increased in NEUROPROTECT coupled with CO augmentation. As described above with *E*_*h*_, we can deduce that *R*_cardiac_ decreased with IV fluid. Improved cardiac function with fluids could be due to decreased afterload or, potentially, increased inotropy [6].

Aneman and colleagues are to be congratulated for their intriguing work. Measures that rely on VRdP should be interpreted together with CO and *R*_VR_; when doing so, novel mechanisms of common interventions (e.g., IV fluids) can be revealed.

## Data Availability

Not applicable.
